# A pathway to sight

**DOI:** 10.7554/eLife.107193

**Published:** 2025-05-21

**Authors:** Katherine J Wert

**Affiliations:** 1 Departments of Ophthalmology and Molecular Biology, University of Texas Southwestern Medical Center Dallas United States; 2 Peter O'Donnell Jr. Brain Institute, University of Texas Southwestern Medical Center Dallas United States; 3 Hamon Center for Regenerative Science and Medicine, University of Texas Southwestern Medical Center Dallas United States

**Keywords:** photoreceptor, glutaminase, metabolism, neurodegeneration, vision, Mouse

## Abstract

The breakdown of glutamine is an important metabolic pathway for the health and survival of rod photoreceptors within the retina.

**Related research article** Goswami MT, Weh E, Subramanya S, Weh KM, Durumutla HB, Hager H, Miller N, Chaudhury S, Andren A, Sajjakulnukit P, Zhang L, Besirli CG, Lyssiotis CA, Wubben TJ. 2024. Glutamine catabolism supports amino acid biosynthesis and suppresses the integrated stress response to promote photoreceptor survival. *eLife* **13**:RP100747. doi: 10.7554/eLife.100747.

Loss of eyesight is often caused by the degeneration of photoreceptors, light-sensing cells in the retina that are critical for processing visual information. Once damaged, these cells cannot regenerate, potentially leading to irreversible visual impairments. Researchers are therefore eager to understand the mechanisms that maintain photoreceptor health and how disruptions to these processes may contribute to cell death. This could lead to the identification of new therapeutic strategies for promoting photoreceptor health and preserving vision.

The retina contains two types of photoreceptors – rods and cones – both of which require large amounts of energy to carry out their roles (Ng et al., 2015). As a result, the retina is one of the most metabolically demanding tissues in the body. Indeed, previous studies have shown that disrupting the production of metabolic nutrients can trigger photoreceptor degeneration (Bowne et al., 2006; Du et al., 2015; Pan et al., 2021).

While glucose metabolism is well-established to be a key source of energy for photoreceptors, it remains unclear which additional metabolic pathways are also required to fuel these cells. Previous studies showed that transcripts for the enzyme glutaminase – which breaks down the amino acid glutamine – are enriched in the retinas of mice and humans (Macosko et al., 2015; Voigt et al., 2020). Now, in eLife, Thomas Wubben and coworkers from the University of Michigan – including Moloy Goswami as first author – report that glutamine metabolism is critical for maintaining the health of rod photoreceptor cells (Goswami et al., 2024).

The team found that glutaminase is present not only within the retinas of mice but specifically in their photoreceptors, supporting the hypothesis that this metabolic enzyme helps generate energy in these cells. Next, Goswami et al. explored whether glutamine metabolism affects retinal health. To do this, they created genetically modified mice that lacked the enzyme glutaminase in their rod cells, the most abundant photoreceptor type in the mouse retina. Goswami et al. found that the mutant mice experienced a gradual loss of photoreceptors, mimicking human retinal degenerative diseases (Figure 1).

**Figure 1.**
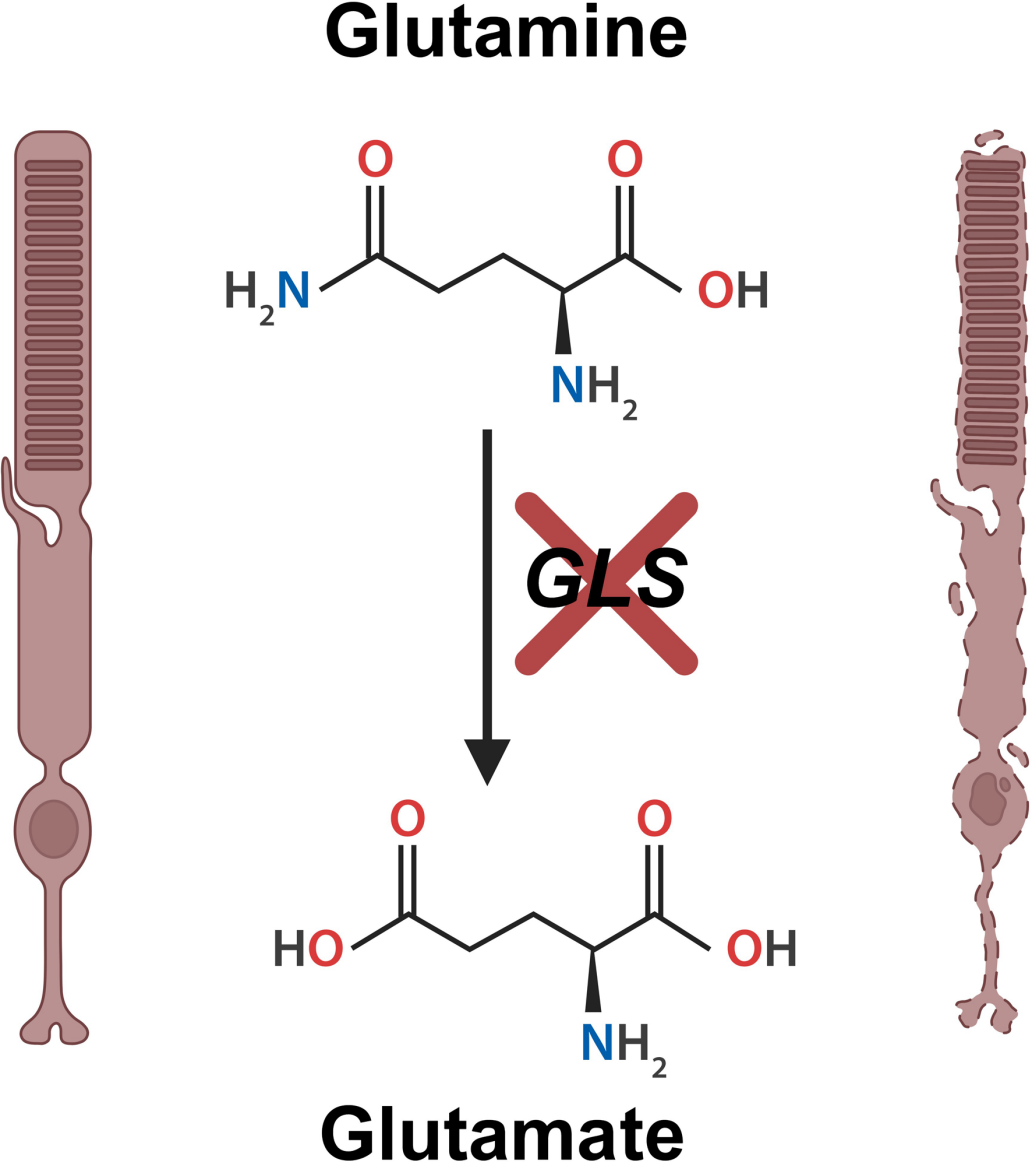
Loss of glutaminase leads to rod photoreceptor cell death. The amino acid glutamine (top middle) is broken down by the enzyme glutaminase (GLS). This leads to the production of the molecule glutamate (bottom middle), which enters the tricarboxylic acid (TCA) cycle where it is further metabolized to generate cellular energy. Goswami et al. found that the loss of GLS (red cross) causes healthy rod photoreceptor cells (left hand side) to degenerate (right hand side), eventually resulting in cell death and potential visual impairments. This figure was created using BioRender.com.

Notably, this photoreceptor degeneration occurred more rapidly than in mouse models lacking key enzymes for glucose metabolism (Weh et al., 2020; Wubben et al., 2017; Daniele et al., 2022; Grenell et al., 2019; Rajala et al., 2023). This indicates that rod photoreceptors do not have a way to compensate for the loss of glutamine metabolism, underscoring its essential role in cell survival.

Goswami et al. then delved deeper into how the glutamine metabolic pathway supports rod photoreceptor cells. Glutamine plays a key role in the tricarboxylic acid (TCA) cycle by supplying intermediate molecules needed to generate energy and synthesize essential components like amino acids. However, Goswami et al. found that deleting glutaminase did not dramatically alter the overall levels of TCA intermediates in the retina, suggesting that reduced activity of the TCA cycle alone is not driving the observed degeneration of photoreceptors. The only change they observed was a reduction in a specific molecule in the TCA cycle called malate.

Goswami et al. discovered that glutamine contributed to the synthesis of the amino acid aspartate, which was reduced in the glutaminase depleted rod cells. To investigate if the synthesis of aspartate is important for photoreceptor health, they provided the mice lacking glutaminase with asparagine, an amino acid that is needed to make aspartate. This slowed down the loss of rod photoreceptors in the mutant mice, indicating that asparagine protects these cells. Further research is needed to learn how asparagine is able to protect photoreceptor cells when they no longer have glutaminase.

Additional experiments revealed that removing glutaminase also activated the integrated stress response, a cellular defense mechanism that regulates protein synthesis and other processes that help cells cope with stress. Goswami et al. found that inhibiting this response slowed down photoreceptor degeneration in the mutant mice.

Taken together, this study provides strong evidence in a live model system that glutamine metabolism is critical for photoreceptor health and preserving vision. Future research is needed to determine how glutamine interacts with the integrated stress response and whether different metabolic pathways are required to preserve the health of cone cells – the other type of photoreceptor in the retina.

The findings of Goswami et al. lay the groundwork for understanding the metabolic needs of photoreceptors, and how metabolic nutrients are shared and used between retinal cells. In the future, these insights could inform the development of new therapies that can prevent or slow vision loss in patients with retinal diseases.
